# Role of Vitamin D in Asthma Control: A Cross-Sectional Study of the Indian Adult Population

**DOI:** 10.7759/cureus.72713

**Published:** 2024-10-30

**Authors:** Shasidharan Krishnan, Gaurav Sikri, Rajay N Bharshankar, Santosh L Wakode, Rekha Jiwane, Rangnath Pundage

**Affiliations:** 1 Physiology, All India Institute of Medical Sciences, Bhopal, Bhopal, IND; 2 Physiology, Armed Forces Medical College, Pune, IND; 3 Physiology, Bharati Vidyapeeth, Pune, IND

**Keywords:** airway remodelling, allergy, asthma control, asthma exacerbations, atopy, hyperesponsiveness, immunomodulation, prevention of infections, sunshine vitamin, vitamin d deficiency

## Abstract

Background

Bronchial asthma is a major health problem both globally and in developing countries like India. This heterogenous disease is characterised by chronic airway inflammation and hyperresponsiveness to various extrinsic and intrinsic stimuli. The innate and adaptive immune mechanisms play an important role in the pathogenesis of asthma. Vitamin D through its immunomodulatory function on innate and acquired immunity can affect asthma control and its exacerbation. In this study, serum levels of vitamin D in bronchial asthma patients were determined, and their correlation with asthma symptom control and exacerbation was studied.

Materials and methods

Serum vitamin D levels of patients diagnosed with bronchial asthma (based on their history and reversible obstruction in spirometry) who reported for follow-up at the Respiratory outpatient department of a tertiary care hospital in Western Maharashtra, India, were tested after obtaining informed consent. A questionnaire was given to assess their asthma control and exacerbations. Based on their symptom control over the past four weeks, they were categorized into well-controlled (WC), partially controlled (PC), and uncontrolled (UC) asthma groups.

Results

Serum vitamin D levels were determined for 70 patients diagnosed with asthma, of which 10 patients fell into the UC group, 28 into the PC group, and 32 into the WC group. Among the 10 patients in the WC group, vitamin D levels were deficient in six (60%) patients, insufficient in two (20%) patients, and normal in two (20%) patients. Among the 28 patients in the PC group, vitamin D levels were deficient in 19 (67%) patients, insufficient in seven (25%) patients, and normal in two (7.1%) patients. Among the 32 patients in the UC group, vitamin D levels were deficient in 17 (53.1%) patients, insufficient in 10 (31.2%) patients, and normal in five (15.6%) patients.

Conclusions

Although vitamin D deficiency was highly prevalent among Indian adult asthma patients, there was no statistically significant relationship between serum vitamin D levels and asthma control.

## Introduction

Bronchial asthma is a major health problem both globally and in developing countries such as India, where its prevalence is approximately 3%, which is nearly 13.09% of the global burden of asthma (262 million people affected by asthma) [1]. Increased industrialization, urbanization, and pollution are contributing to the increased burden of bronchial asthma on India’s healthcare system.

Asthma is a heterogeneous disease usually characterized by chronic airway inflammation. As per the Global Initiative (GINA) 2019 guidelines, asthma is defined as a history of respiratory symptoms such as wheezing (i.e., noisy breathing), shortness of breath, chest tightness, and cough, which vary over time and in intensity, together with variable expiratory airflow limitation. Airflow limitation may later become persistent. In patients with respiratory symptoms, greater variation in lung function increases the likelihood of diagnosis of asthma. A postbronchodilator increase in forced expiratory volume in one second (FEV1) and/or forced vital capacity (FVC) of >10% from baseline is diagnostic of asthma. If a spirometer is not available, a change in peak expiratory flow rate of at least 20% is accepted as being consistent with asthma [2].

Asthma is a chronic condition in which there is persistent inflammation and hyperresponsiveness of the airways to various intrinsic and extrinsic stimuli. Symptoms of asthma are generally triggered by viral infections, cold, exercise, allergen exposure, changes in weather, irritant smoke, or strong smell [2]. In asthmatics, the extent to which asthma symptoms are controlled or reduced by treatment is called asthma control. Assessment of asthma control is based on two factors: symptom control and risk of future adverse outcome in the form of acute exacerbations. Based on respiratory symptoms, nocturnal awakenings, and pulmonary function tests (i.e., FEV1, FVC, FEV1/FVC ratio) over the past four weeks, asthma can be categorized as well-controlled (WC), partially controlled (PC), or uncontrolled (UC) [3,4].

Pathogenesis of asthma involves both the innate and adaptive immune mechanisms. On exposure to known allergens or to various nonspecific stimuli, there is an initiation of a cascade of cellular activation events in the airways resulting in both acute and chronic inflammatory processes, which are mediated by a complex and integrated release of cytokines and other mediators [5]. The release of mediators can alter airway smooth muscle tone and responsiveness, produce mucus hypersecretion, and damage the airway epithelium. These pathologic events cause structural and functional abnormality in the airway. There are two types of response to allergens: immediate asthmatic response, where symptoms develop immediately after allergen exposure, and late asthmatic response, where symptoms develop four to six hours after allergen exposure.

Vitamin D is a prohormone cholecalciferol that is synthesized in the Malpighian (stratum basale, stratum spinosum) layer of the skin from 7-dehydrocholesterol on exposure to UV-B rays of sunlight for at least 10-30 min for three to four days per week between 11 am to 2 pm, or obtained via diet. Vitamin D is metabolized in the liver to form 25-hydroxyvitamin D [25(OH) Vit D], which is the major circulating biomarker of vitamin D. 25(OH) Vit D is then metabolized in the kidneys by 25-hydroxyvitamin D-1-alpha hydroxylase to its active metabolite, 1-alpha,25-dihydroxyvitamin D [1,25(OH)2Vit D] or calcitriol.

Traditionally, it was thought that vitamin D was only required for maintaining normal blood levels of calcium and phosphate, which, in turn, is required for normal bone mineralization, muscle contraction, nerve conduction, and general cellular functions of all cells. Recent studies have shown that vitamin D is increasingly associated with other systems, such as cardiovascular health, pulmonary function, autoimmune conditions, autonomic dysfunctions, and different cancers [6]. Some studies have also shown that low levels of vitamin D are seen in patients with poor asthma control, reduced lung function, reduced glucocorticoid response, more frequent exacerbations, resulting in increased steroid use, and increased susceptibility to airway remodeling and allergic reaction [6,7]. As hyperresponsiveness of the immune system plays an important role in the pathogenesis of asthma, vitamin D through its immunomodulatory function helps in asthma control and reducing exacerbation.

There are very few studies on the effect of vitamin D levels in bronchial asthma patients among the Indian adult population. Vitamin D levels in healthy individuals and asthmatic patients should be compared, and adequate, insufficient, and deficient vitamin D levels must be identified.

The aim of this study was to determine whether there is a relationship between asthma and vitamin D levels, and, if present, how vitamin D levels vary among WC, PC, and UC asthma patient groups in the Indian adult population. The secondary objective of the study was to investigate the correlation between the vitamin D levels and pulmonary function of asthma patients using spirometry.

## Materials and methods

Study design

This was a cross-sectional observational study that was carried out over a period of two years, from June 1, 2019, to October 30, 2021. Institutional Ethical Committee, Armed Forces Medical College issued approval IEC/04/2019.

Study population

A total of 70 patients diagnosed with bronchial asthma (male and female) who were receiving regular follow-up at the Respiratory Medicine outpatient department of a tertiary care hospital (Armed Forces Medical College and Army Institute of Cardiothoracic Sciences) in Western Maharashtra, India, participated in the study. The participants were between 18 and 65 years of age. Informed consent was obtained from all participants before enrolling them in the study. Participation of individuals in the study was voluntary, and they were free to opt out of the study at any time.

Inclusion criteria

Patients of either sex were included in the study if they were between 18 and 65 years of age, were diagnosed with bronchial asthma, and consented to undergo blood examination to determine their serum vitamin D levels.

Exclusion criteria

Patients with other respiratory illnesses (e.g., tuberculosis, bronchiectasis, chronic obstructive pulmonary disease, empyema, interstitial lung disease); bronchial asthma patients with known co-morbidities such as ischemic heart disease, kidney disease, liver disease, and extensive skin disorders (e.g., vitiligo, albinism, psoriasis, autoimmune) in whom vitamin D levels would be altered; pregnant women, psychiatric patients, bedridden patients, patients on long-term corticosteroid treatment (>30 days), and those receiving vitamin D and calcium supplementation for various causes were excluded from the study.

Methodology

The patients were diagnosed with bronchial asthma based on their history and reversible obstruction in spirometry. Serum vitamin D levels were tested after receiving informed consent from each patient. The history of each patient was taken using the questionnaire given in Appendix 1, which covered symptom frequency in the past four weeks, daytime symptoms occurring more than twice per week, night awakenings, need for reliever use more than twice per week, and limitation of activities. Based on these key questions, they were categorized into partially controlled, well-controlled and uncontrolled asthmatics as per GINA guidelines.

A venous blood sample collected in a gold-top (serum separator-SST) tube was used to estimate vitamin D levels using a Vidas automated immuno-analyzer (bioMérieux, Marcy-l'Etoile, France). Serum vitamin D levels of >30 ng/mL, 21-29 ng/mL, and <20 ng/mL were considered normal, insufficient, and deficient, respectively, and levels of >100 ng/mL were considered potentially toxic [8].

Only 11 of the 70 patients could be subjected to spirometry for studying the correlation between vitamin D levels and pulmonary function among asthma patients, as the study was carried out between 2019 and 2022, during which the COVID-19 pandemic was at its peak, and all aerosol-generating procedures were disallowed. The patients were asked to stop short-acting and long-acting bronchodilators at least six hours and 24 hours prior to testing, respectively. They were also advised to have a light breakfast two hours prior to testing, and to avoid smoking, alcohol consumption, and exercise for at least four hours before the test. Spirometry testing was carried out between 8:00 am and 9:00 am. A dynamic spirometry manoeuvre in the form of forced inspiration followed by a blast of expiration was performed at an ambient temperature [4].

FVC, FEV1, and their ratio (FEV1/FVC) were recorded to assess lung function. A FEV1/FVC of ≤70% was the demarcation point for airway obstruction during dynamic spirometry. A change of ≥10% in either FVC or FEV1 after administration of bronchodilator levo-salbutamol 50 mcg (four doses one minute apart each and the test performed after 15 min) suggested reversible airway obstruction [4].

Sample size calculation

The sample size was calculated to ascertain a confidence interval (CI) 95% for the mean, considering mean score as 19.88 and 9.60 (SD) from previous similar studies in adult population. Sample size for estimating CI for mean (absolute error of margin with no finite correction) (1-α)- 0.9SD-9.60, D-1.50, N=157. Thus, the required sample size was calculated to be 157 [9]. Due to the COVID-19 pandemic and resulting lockdowns, data were obtained for only 70 participants. Detailed data is shown in Appendix 2.

Statistical analysis

One-way ANOVA was used to determine any differences in vitamin D levels among the three groups (WC, PC, and UC) after testing for homogeneity of variance. Correlation between quantitative variables (i.e., age and asthma duration) and vitamin D levels was determined using Pearson’s correlation coefficient test. IBM SPSS software version 20.0 (IBM Corp., Armonk, NY, USA) was used for data analysis.

## Results

Total serum vitamin D levels were estimated for 70 patients, of which 41 (59%) were female and 29 (41%) were male. Serum vitamin D levels were either deficient or insufficient in 61 (87%) patients with 42 (60%) and 19 (27%) patients having deficient and insufficient vitamin D levels, respectively.

Based on the frequency of symptoms experienced in the past four weeks (i.e., daytime symptoms more than twice per week, night awakenings, need for reliever use more than twice per week, and limitation of activities), the patients were categorized into WC (none of the above-mentioned symptoms), PC (one to two of the above-mentioned symptoms), and UC (three to four of the above-mentioned symptoms) groups [1,2]. Accordingly 10 out of the 70 patients were categorised into WC, 28 were categorised into PC and the remaining 32 were categorised into UC asthma groups.

**Figure 1:**
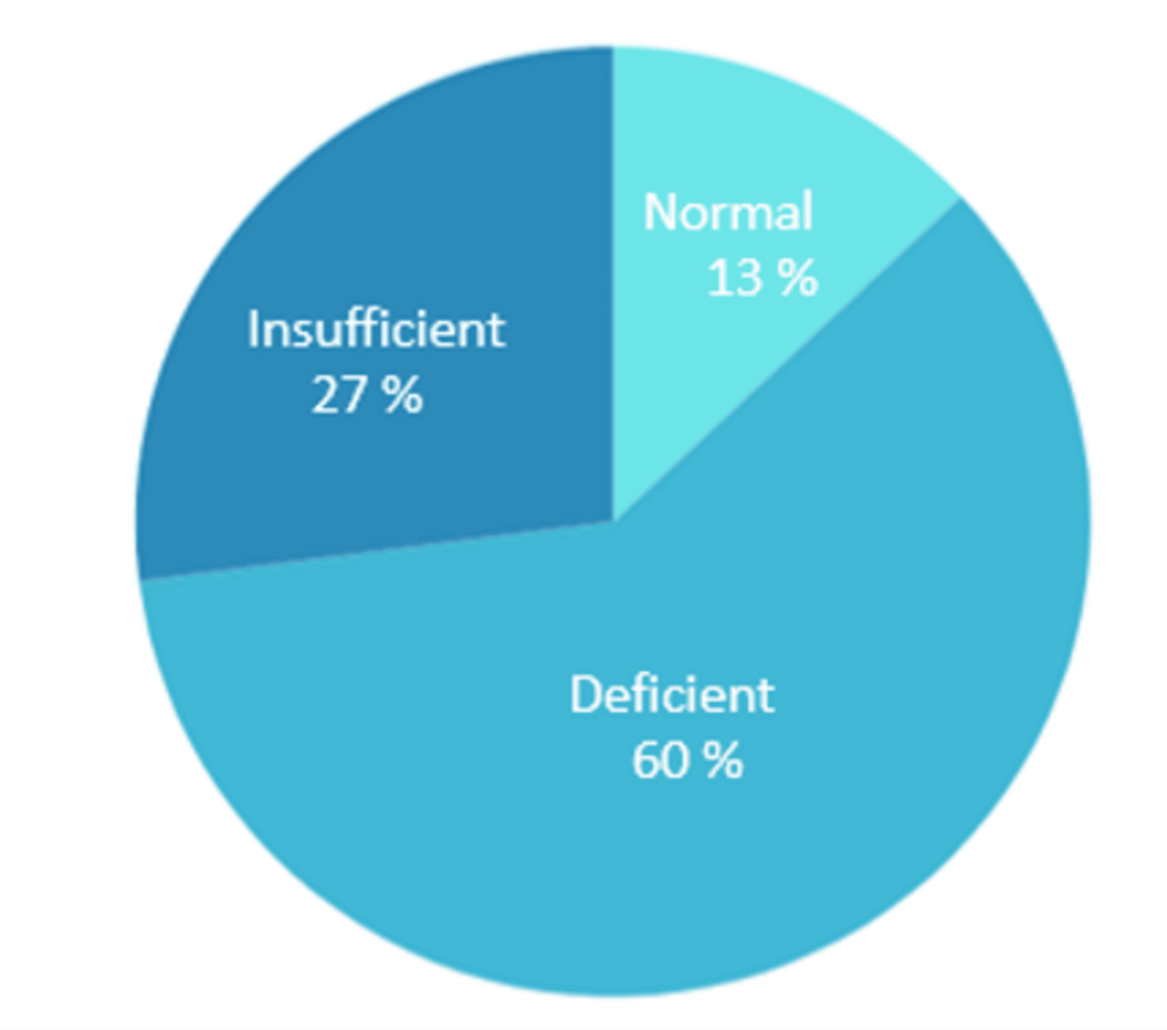
Percentage distribution of asthma patients based on their serum vitamin D levels. vitamin D levels of >30 ng/mL, 21–29 ng/mL, and <20 ng/mL were considered normal, insufficient, and deficient, respectively

Among the 10 patients in the WC group, vitamin D levels were deficient in six (60%) patients, insufficient in two (20%) patients, and normal in two (20%) patients. Among the 28 patients in the PC group, vitamin D levels were deficient in 19 (67%) patients, insufficient in seven (25%) patients, and normal in two (7.1%) patients. Among the 32 patients in the UC group, vitamin D levels were deficient in 17 (53.1%) patients, insufficient in 10 (31.2%) patients, and normal in five (15.6%) patients, as shown in Table 1 and Figure 1.

**Table 1:** Vitamin D status among different asthma control groups WC: well controlled, PC: partially controlled, UC: uncontrolled. Data are presented as frequencies and percentages.

Asthma Control Group	Normal > 30 ng/mL	Insufficient 20–29 ng/mL	Deficient <20 ng/mL	Total
WC	2 (20%)	2 (20%)	6 (60%)	10
PC	2 (7.1%)	7 (25%)	19(67%)	28
UC	5 (15.6%)	10 (31.2%)	17(53%)	32
Total	9 (13%)	19 (27%)	42 (60%)	70

**Figure 2:**
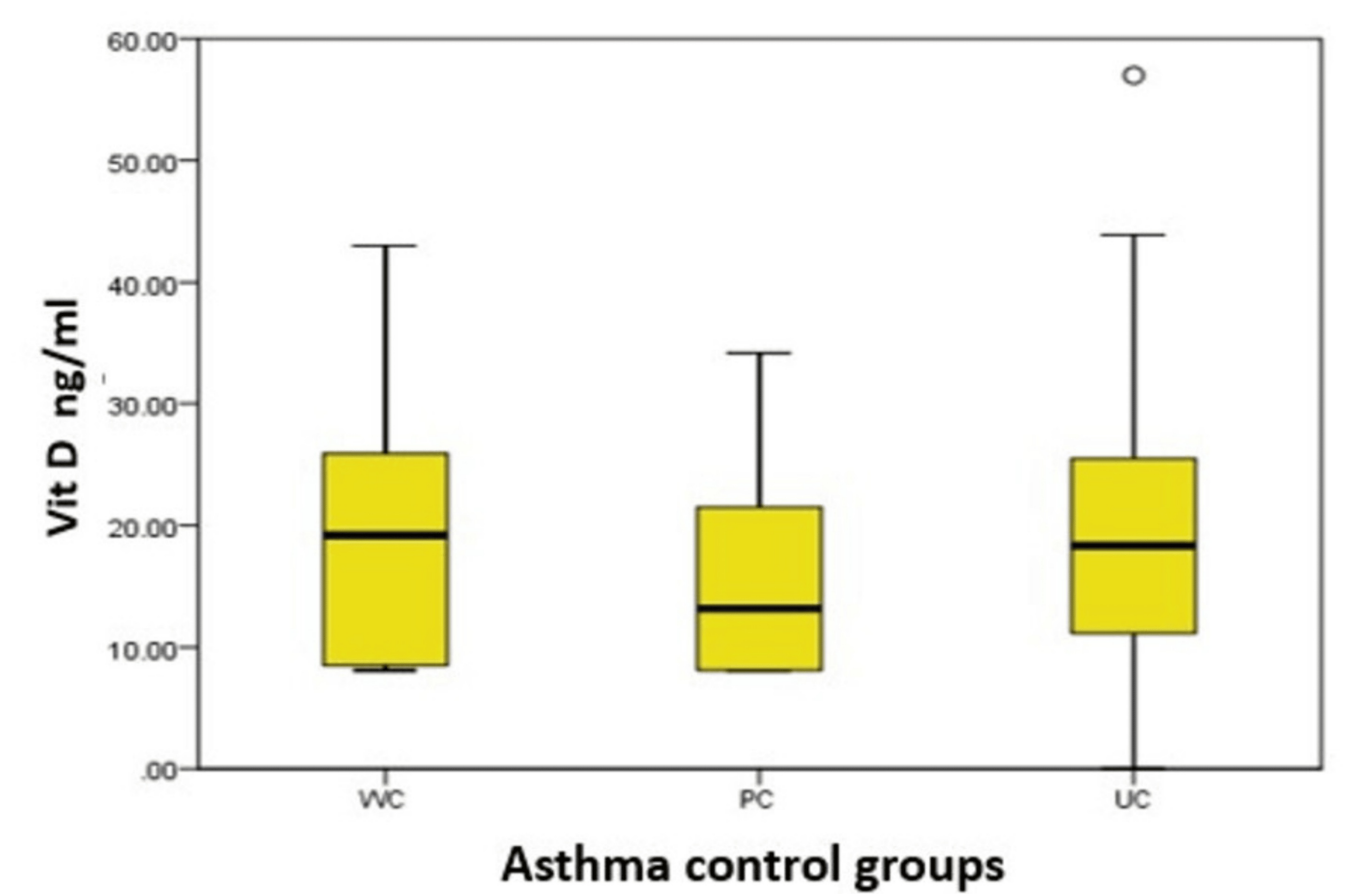
Vitamin D levels among asthma control groups.

ANOVA revealed no significant difference in vitamin D levels between the three asthma control groups (p=0.180), with mean vitamin D levels of 21.83 (SD=12.9), 15.87(SD=8.32), and 20.05 (SD=11.17) in the WC, PC, and UC groups, respectively, as shown in Table 2, Table 3, and Figure 2.

**Table 2:** Mean vitamin D levels among WC, PC, UC asthma control groups WC: well controlled, PC: partially controlled, UC: uncontrolled.

Asthma control group	n	Mean ± Standard Deviation
WC	10	21.83 ± 12.90
PC	28	15.87 ± 8.32
UC	32	20.05 ± 11.17
Total	70	18.64 ± 10.51

**Table 3:** ANOVA test comparing the vitamin D levels between and within different asthma control groups

Vitamin D levels	Sum of Squares	df	Mean Square	F	Sig.
Between Groups	379.79	2	189.89	1.75	0.180
Within Group	7242.93	67	108.10	----	-----
Total	7622.73	69	NA	-----	-----

**Figure 3:**
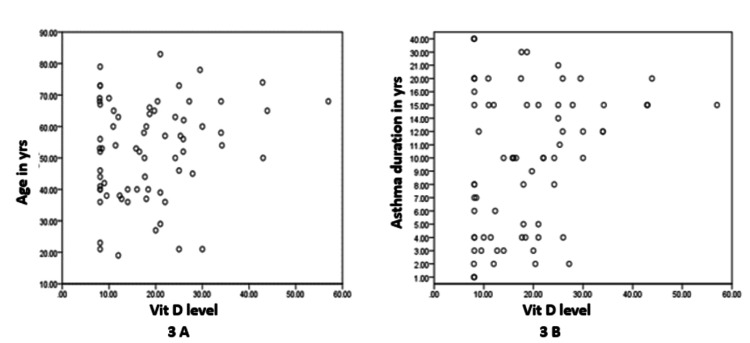
Correlation between age (A) and duration of asthma (B) with serum vitamin D levels.

Correlation analyses found insignificant and weak correlations between patient age and vitamin D level, with r = 0.204 (p = 0.09), and between asthma duration and vitamin D level, with r = 0.11 (p = 0.37), as shown in Table 4 and Figures 3A, 3B.

**Table 4:** Correlation between age and duration of asthma with serum Vitamin D levels. *Correlation is significant at the 0.05 level (two-tailed).

Variables		Vitamin D level	Asthma duration
Age in years	r	0.204	0.244
	Significance (two-tailed)	0.093	0.42
	N	70	70
Asthma duration in years	r	0.109	-----
	Significance (two-tailed)	0.370	-----
	N	70	-----

## Discussion

In this study, we measured the total serum vitamin D levels of 70 asthma patients, of which 59% were female and 41% were male. The mean age of the participants was 52.1±15.58 years. We found no significant difference in age between the three asthma control groups (WC, PC, and UC) (p = 0.76). Vitamin D levels were deficient (<20 ng/mL) in 42 (60%) patients, insufficient (20-29 ng/mL) in 19 (27%) patients, and normal (>30 ng/mL) in nine (12.85%) patients. No significant difference in vitamin D levels was observed based on gender (p = 0.713). Furthermore, no significant difference in vitamin D levels was observed between the three asthma control groups (p = 0.180), with mean serum vitamin D levels of 21.83 ± 12.9 ng/mL in the WC group, 15.87 ± 8.32 ng/mL in the PC group, and 20.05 ± 11.17 ng/mL in the UC group, as seen in Tables 2, 3.

In Egypt, Shahin et al. studied vitamin D levels in 70 asthma patients and 20 healthy volunteers in a similar setting, showing that serum vitamin D levels were significantly lower in asthma patients (19.88 ± 9.6 ng/mL) as compared with the healthy control group (33.5 ± 6.1 ng/mL). In addition, compared to the controlled asthma group (20.5 ± 7.5 ng/mL), serum vitamin D levels were significantly lower in uncontrolled asthma patients (10.5 ± 5.2 ng/ml) [9]. However, the mean age of the participants (34.4 ± 6 years) and inclusion of a control group were marked differences compared to the current study. Another Egyptian study by Samaha et al. of 55 participants (mean age = 37.6 ± 8.9 years) comprising 40 asthma patients and 15 healthy controls reported a positive correlation between vitamin D status and asthma control [6].

The relationship between vitamin D levels and asthma control has also been studied among children. Chinellato et al. reported that vitamin D deficiency is common in asthmatic children living in Italy [10], where lower levels of vitamin D were associated with reduced asthma control. Children with well-controlled asthma (median [Q1; Q3])=22.2 [16.3; 25.4]) had higher serum vitamin D levels compared to children with partially controlled (17.8 [11.8; 22.1]) and un-controlled asthma (18.1 [15.0; 18.5]), and this difference was significant (p = 0.023).

The paediatric age group also was analysed retrospectively by Searing et al. in their study of 100 asthmatic children, reporting prevalences of vitamin D insufficiency (<30 ng/mL) and deficiency (<20 ng/mL) of 47% and 17%, respectively, among childhood asthma patients [11]. They also reported that vitamin D supplementation might potentiate the anti-inflammatory function of corticosteroids in asthmatic patients and thereby improve asthma control.

A study by Gupta et al. found that reduced vitamin D levels in severe therapy-resistant asthma were associated with lower lung function, poorer asthma control, increased medication use, and asthma exacerbations [12]. Vitamin D levels also were correlated with asthma severity in a study by Zayadneh et al. in Jordan [13]. Among 98 asthmatic children, vitamin D levels were deficient and insufficient in 41 (41.8%) and 34 (34.7%) children, respectively, and only 23 (23.5%) had sufficient vitamin D levels. They also found that vitamin D deficiency was higher in children with severe and moderate persistent asthma compared to children with intermittent or mild asthma.

The relationship between asthma severity and vitamin D deficiency was further explored in a randomized control trial study of 42 asthma patients by Menon et al. in India, where there was a significant improvement in asthma control after supplementation with vitamin D (p < 0.05) [14]. In a cross-sectional case control study on 120 participants (60 cases and 60 controls) in India, Shaik et al. found that asthma patients (44.9 ± 12 nmol/L) had significantly lower vitamin D compared to controls (86 ± 13.3 nmol/L) (p = 0.001) [15]. In contrast, few studies observed no association between asthma and vitamin D levels, and others observed an increased incidence of allergy/asthma with higher vitamin D levels [16-18].

In a prospective study conducted over five years on 4999 Danish adults, Thuesen et al. reported contrasting results, suggesting no role of vitamin D in the development of asthma or allergy [16]. However, in their study, diagnosis of asthma was based on a questionnaire, patient ages ranged from 30 to 60 years, serum vitamin D levels were measured using high-performance liquid chromatography, lung function was assessed by spirometry, and no information on supplementation of vitamin D was available. Similarly, in a prospective study of a Norwegian population, Brumpton et al. did not find any strong evidence that adult patients with asthma were at greater risk of low serum vitamin D level, or that being a non-user of vitamin D supplements was associated with an increased risk of poorly controlled asthma [17]. There were a number of shortcomings in their study: participants were assigned to the asthmatic group based on a questionnaire, they did not have complete data on vitamin D or any other supplements, the study included only female patients, all confounders were not matched, and the sample size and power of the study were limited, as conceded by the authors themselves.

Jolliffe et al. performed a multicentric study on 297 adult asthma patients in the UK to determine whether vitamin D status was independently associated with Asthma Control Test score [18]. No association was found between vitamin D status and markers of asthma severity or control. Kavitha et al., in their study on 105 children (5-15 years of age) with asthma in India, found that there was no significant difference (p = 0.24) in serum vitamin D levels among asthma control groups (WC, PC, UC) [19], with median (interquartile range) levels of 9.0 (6.75-15) ng/mL in the WC group, 10 (6.25-14.75) ng/mL in the PC group, and 8.0 (5-10) ng/mL in the UC group. They did not observe any association between serum vitamin D levels and level of asthma control.

The findings of the above-mentioned paediatric studies are consistent with those of our study among the adult Indian population: vitamin D deficiency is prevalent in asthma patients. However, no correlation is seen between serum vitamin D levels and asthma control in both studies.

In a case control study of children with bronchial asthma aged six to 12 years by Anand et al., a high prevalence of vitamin D deficiency (<20 ng/mL) was found among asthmatic children as compared to healthy volunteers (p < 0.001). A multivariate analysis adjusting for age, body mass index, and sex, the relationship between low vitamin D levels and prevalence of asthma further increased. However, no association was found between vitamin D levels and level of asthma control [20].

The findings of our study are consistent with those of previous studies, where a significant number (87%) of asthma patients had vitamin D deficiency/insufficiency (95% CI: 79%-95%). However, there was no statistically significant difference (p = 0.180) in vitamin D levels among the different asthma control groups (WC, PC, UC). This study confirms that vitamin D deficiency is highly prevalent in Indian adult asthma patients. Further randomized controlled trials are required to determine the benefits of vitamin D supplementation in asthma patients.

Strengths of the study

This study was conducted in the Indian adult population; there are very few similar studies in adult asthma patients that determined the effect of serum vitamin D levels on asthma control. There was no significant difference in the demographic parameters of the patients in different asthma control groups and they were comparable. Early diagnosis of vitamin D deficiency among asthma patients was possible to prevent other complications of vitamin D deficiency. 

Limitations of the study

All patients could not be subjected to spirometry due to the ongoing COVID-19 pandemic. Therefore, classification of asthma control groups was only based on the questionnaire results, with possibility of recall bias. Furthermore, vitamin D levels of the healthy Indian population were not measured nor compared.

## Conclusions

Although vitamin D deficiency is highly prevalent among Indian adult asthma patients, we found no statistically significant relationship between serum vitamin D levels and asthma control. Further prospective analytical studies comparing the vitamin D levels of asthma patients with those of healthy individuals acting as controls may be required to determine the exact role of vitamin D levels in the pathogenesis of asthma. Randomized controlled trials with a larger sample size dividing asthma patients into two groups, with one group being supplemented with vitamin D and the other group receiving a placebo, may be ideal in recognizing the exact role of vitamin D in the control of asthma and its exacerbation.

## Figures and Tables

**Figure 4:**
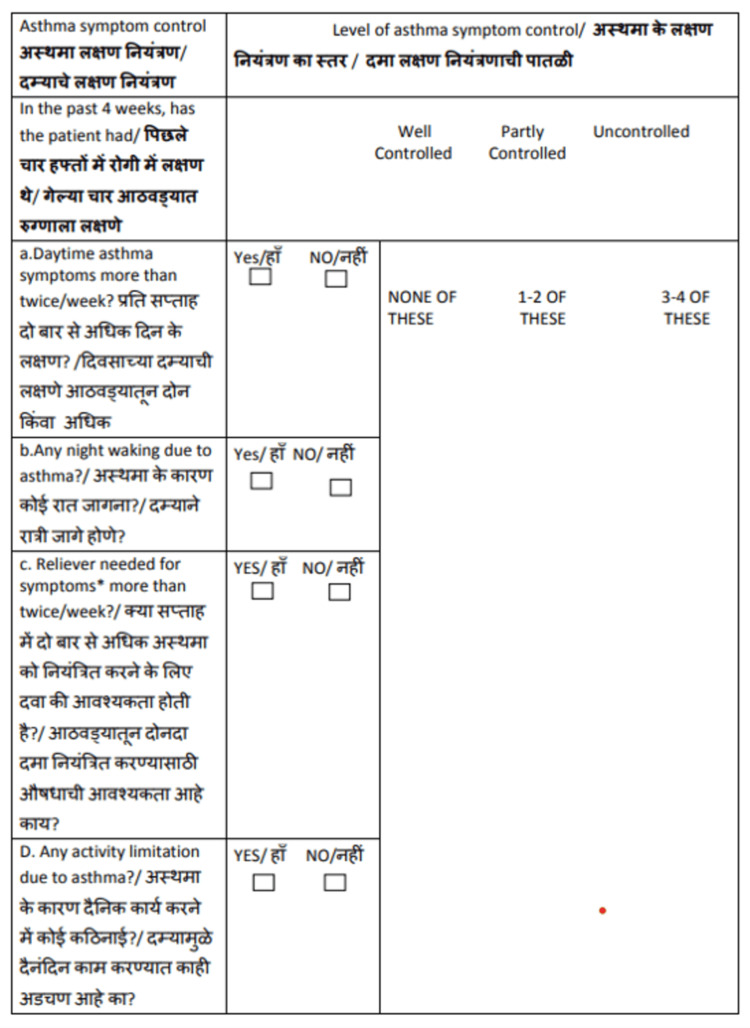
Questionnaire to categorise asthma patients into WC, PC, UC groups WC: well controlled, PC: partially controlled, UC: uncontrolled

## Appendices

Appendix 1

Appendix 2

**Table 5:** Data sheet showing demographics, asthma duration, asthma control category, vitamin D levels and pulmonary function test of participants *Pulmonary function test could be done only for 11 participants due to covid pandemic. FEV1: forced expiratory volume in one second, FVC: forced vital capacity, WC: well controlled, PC: partially controlled, UC: uncontrolled

S No	Age yrs	Gender	Asthma duration yrs	Asthma control	Vitamin D levels	Pre FEV1 /FVC	Pre FVC	pre FEV1	Post BD FVC	PB FEV1	Predicted FVC
1	65	F	9	WC	19.7						
2	53	F	7	WC	8.5						
3	21	F	6	WC	8.1						
4	52	F	12	WC	25.9						
5	40	F	4	WC	18.4						
6	66	F	30	WC	18.7						
7	50	F	15	WC	43						
8	40	F	20	WC	8.1						
9	74	M	15	WC	42.9						
10	46	M	15	WC	25						
11	44	F	4	PC	17.7	57.18	1.19	0.69	1.38	0.86	2.72
12	44	F	7	PC	8.1						
13	50	F	10	PC	24.2						
14	36	F	10	PC	22						
15	40	F	3	PC	14						
16	65	F	15	PC	11						
17	56	F	20	PC	25.9						
18	68	F	1	PC	8						
19	41	F	20	PC	8.1	73	2.44	1.78			2.67
20	63	F	15	PC	12						
21	39	F	5	PC	21	69.28	2.73	1.89	2.44	2.17	2.93
22	38	F	6	PC	12.3						
23	23	F	8	PC	8.1						
24	36	F	10	PC	14						
25	57	M	11	PC	25.3						
26	53	M	10	PC	15.8						
27	83	M	15	PC	21						
28	38	M	3	PC	9.5						
29	69	M	4	PC	10						
30	78	M	20	PC	29.5						
31	58	M	20	PC	17.5						
32	56	M	15	PC	8.1						
33	68	M	12	PC	34	68.5	1.48	1.08	1.91	1.41	3.04
34	54	M	15	PC	34.19						
35	36	M	8	PC	8.1						
36	40	M	16	PC	8.1						
37	42	M	12	PC	9						
38	52	M	20	PC	8.1						
39	52	F	10	UC	16.5	52.15	1.56	0.81	1.8	1.01	2.76
40	19	F	2	UC	12						
41	68	F	15	UC	57						
42	37	F	3	UC	12.7						
43	50	F	30	UC	17.6						
44	46	F	3	UC	8.1						
45	60	F	12	UC	30						
46	65	F	20	UC	43.9	65..2	1.12	0.73	1.1	0.74	2.29
47	68	F	2	UC	27.2						
48	54	F	4	UC	11.4						
49	58	F	12	UC	34						
50	53	F	40	UC	8	74.04	2.24	1.66	2.22	1.6	3.52
51	57	F	10	UC	22						
52	73	F	4	UC	8.1	55.24	1.05	0.64	1.19	0.75	46.10%
53	64	F	15	UC	18.7						
54	37	F	8	UC	18						
55	45	F	15	UC	27.9						
56	67	F	4	UC	8.1	61.83	1.86	1.15	2.05	1.32	2.25
57	69	F	2	UC	8						
58	73	M	1	UC	8.1						
59	21	M	10	UC	30						
60	21	M	14	UC	25						
61	63	M	8	UC	24.2						
62	60	M	20	UC	10.9						
63	68	M	2	UC	20.4	79.2	1.73	1.39	1.77	1.48	2.93
64	40	M	10	UC	16						
65	79	M	40	UC	8.1						
66	73	M	21	UC	25	55.5	1.17	2.11	2.18	1.4	
67	62	M	4	UC	26						
68	27	M	3	UC	20						
69	60	M	5	UC	18						
70	29	M	4	UC	21						
